# Public Awareness as a Tool for Improving Community Health: A Systematic Review of Communication and Community-Based Interventions

**DOI:** 10.7759/cureus.107930

**Published:** 2026-04-28

**Authors:** Shrilekha, Regina Wahengbam, Biswabinod Sanfui, Pallavi Mishra, Komal Pukur Thekdi, Renuka Chawre, Rajat Gupta

**Affiliations:** 1 Department of Community Medicine, World College of Medical Sciences and Research, Jhajjar, IND; 2 Department of Community Medicine, K D Medical College Hospital and Research Centre, Mathura, IND; 3 Department of Community Medicine, Dr. N. D. Desai Faculty of Medical Sciences and Research, Dharmsinh Desai University, Nadiad, IND; 4 Department of Swasthavritta and Yoga, Maharashtra University of Health Sciences, Nashik, IND; 5 Department of Community Medicine, Bhausaheb Mulak Ayurved College and Research Hospital, Nagpur, IND

**Keywords:** community engagement, health communication, health literacy, mass media, public awareness

## Abstract

Health literacy influences health behaviors by shaping individuals’ ability to access and apply health information. Media and community-based strategies are increasingly used to improve awareness; however, comparative evidence on their effectiveness, particularly in integrated forms, remains limited. This systematic review synthesizes evidence on communication and community engagement interventions and their impact on health literacy, awareness, and behavior. A structured search was conducted across PubMed, Scopus, and Google Scholar for English-language studies published between 2015 and 2025. Following screening and eligibility assessment, 10 studies were included, comprising randomized controlled trials, observational studies, and campaign evaluations. Data were extracted using a standardized approach and analyzed through narrative synthesis, with interventions categorized as media/digital or educational/community-based. Media and digital interventions were associated with improvements in awareness and risk perception, whereas educational and community-based approaches were linked to sustained behavior change and increased participation. Integrated interventions showed greater overall effectiveness compared to single-component strategies. These findings support the use of combined communication and participatory approaches to achieve both immediate and sustained public health outcomes.

## Introduction and background

Health literacy is a critical determinant of population health, defined as the ability of individuals to access, understand, appraise, and apply health information to make informed health decisions, consistent with established frameworks such as Nutbeam’s model of functional, interactive, and critical health literacy [1]. It plays a key role in promoting preventive behaviors, improving health outcomes, and reducing health inequalities across populations [2]. Health-literate individuals are more likely to adopt healthy lifestyles, adhere to medical advice, and effectively utilize healthcare services.

Efforts to enhance health literacy have increasingly relied on structured communication strategies, particularly in response to the growing burden of infectious and chronic diseases [3]. At a foundational level, mass media have historically served as a primary channel for disseminating health information to large populations. Traditional platforms such as television and radio have been effective in shaping public perceptions and influencing health behaviors through consistent and repetitive messaging [4]. Building on this foundation, digital communication has expanded the reach, speed, and accessibility of health information. Within established health literacy frameworks, online platforms support not only access to information but also individuals’ ability to understand, appraise, and apply health knowledge in real time, especially during public health emergencies such as disease outbreaks and pandemics [5,6].

Within the broader domain of digital communication, social media represents a more interactive extension of these approaches. Unlike traditional media, social media facilitates two-way communication, enabling users to engage with content, share information within networks, and participate in health-related discussions [7]. This interactivity enhances awareness and knowledge dissemination, particularly when information is tailored and context-specific [8,9]. However, the effectiveness of social media interventions depends on the credibility, accuracy, and relevance of the information shared.

While communication strategies are effective in raising awareness, translating this awareness into sustained behavior change often requires more structured and interactive approaches. Educational interventions address this need by focusing on knowledge acquisition and skill development. Methods such as video-assisted learning and peer education simplify complex health information and improve comprehension, particularly among younger populations [10]. These approaches help bridge knowledge gaps and strengthen individuals’ capacity to make informed health decisions [11]. Public health campaigns further extend these strategies at the population level by combining messaging with behavioral framing, contributing to measurable changes in attitudes and risk perceptions, particularly in areas such as smoking prevention [5,12].

Beyond communication and education, community engagement represents a participatory approach that emphasizes local involvement, empowerment, and context-specific solutions. Community-based strategies enable individuals and groups to actively participate in identifying and addressing health challenges, making them particularly effective in settings where health behaviors are shaped by social and cultural factors [3]. Interventions such as image-based awareness campaigns and structured models sucha s SALT (Stimulate, Appreciate, Learn, Transfer) have demonstrated improved engagement, increased uptake of health services, and potential for sustained behavior change through community ownership [9,13,14]. While mass media and digital platforms are effective in disseminating information at scale, their ability to produce sustained behavioral change is limited without more interactive and participatory approaches [15].

Despite the availability of these diverse approaches, a key challenge remains in translating increased awareness into sustained behavioral change. The effectiveness of interventions is influenced by socio-demographic factors, resource availability, and cultural context, limiting the generalizability of findings. Existing literature, including prior systematic reviews, has primarily examined individual interventions such as mass media campaigns or educational programs, often focusing on short-term outcomes within specific contexts. Although these studies demonstrate improvements in knowledge and risk perception, they provide limited insight into long-term behavioral change and rarely evaluate integrated approaches that combine communication strategies with community engagement. Furthermore, heterogeneity in study designs and outcome measures restricts comparability and synthesis across studies. Given the increasing complexity of public health challenges and the expanding role of digital and participatory strategies, a comprehensive synthesis that evaluates both communication-based and community engagement interventions, individually and in combination, is necessary to better understand their collective effectiveness and inform future public health practice.

Objective of the review

This review aimed to synthesize available evidence on communication-based and community engagement interventions for enhancing health literacy, awareness, and health-related behaviors. It sought to examine the extent, consistency, and nature of evidence regarding the effectiveness of media, educational, and participatory strategies across different public health contexts, with particular attention to variations in study design, outcome measures, and implementation settings. In addition, the review aimed to identify gaps in existing literature, particularly the limited evidence on integrated approaches that combine communication and community engagement, and their impact on sustained health literacy and long-term behavior change.

## Review

Methodology

Study Design

The systematic review study aimed at assessing the efficacy of communication-based and community engagement interventions to enhance the health awareness and behavior of the people. The review was conducted in accordance with the Preferred Reporting Items for Systematic Reviews and Meta-Analyses (PRISMA) guidelines to ensure methodological rigor and transparency. A predefined protocol guided the review process; however, it was not prospectively registered in the PROSPERO database. Studies that had various research designs, which include randomized controlled trials, quasi-experimental studies, observational studies, and campaign evaluations, were included in the review. This was intended to be a synthesis of existing evidence based on different methods. The research method was aimed at revealing trends and making reasonable conclusions based on the current literature instead of creating primary data, which would guarantee a full concept of the effectiveness of the intervention.

Search Strategy

A comprehensive search strategy was conducted across PubMed, Scopus, and Google Scholar to identify relevant studies published in English between January 2015 and December 2025. The search utilized predefined keywords and controlled vocabulary where applicable, including “health literacy”, “public health communication”, “mass media”, “social media”, and “community engagement”. These terms were systematically combined using Boolean operators: “AND” was used to link different concepts (e.g., “health literacy AND media interventions”), while “OR” was used to include synonymous terms (e.g., “mass media OR social media”). Only peer-reviewed articles were included, and grey literature (e.g., reports, theses, conference proceedings) was excluded, which may introduce potential publication bias. To enhance coverage, backward and forward citation tracking was performed using Google Scholar to identify additional relevant studies from reference lists and citing articles. This structured approach was designed to ensure comprehensive retrieval of studies across diverse public health contexts while maintaining methodological transparency and reproducibility.

Eligibility Criteria

Studies were included if they evaluated communication-based or community engagement interventions aimed at improving health literacy, awareness, or health-related behaviors among individuals or communities across diverse settings. Eligible interventions included mass media campaigns, social media and digital communication strategies, educational programs (e.g., video-assisted learning and peer education), and participatory community-based approaches. Studies reporting measurable outcomes, such as changes in health literacy, knowledge, attitudes, risk perception, behavioral intentions, or actual health behaviors, were considered for inclusion. Both experimental and observational study designs, including randomized controlled trials, quasi-experimental studies, cross-sectional studies, cohort studies, and campaign evaluations, were included to capture a broad range of evidence. Studies with or without comparison groups were eligible, provided that they reported quantifiable outcomes. Studies were excluded if they were not published in English, did not report relevant outcome measures, or did not focus on the specified intervention types. Non-primary research articles, including reviews, editorials, commentaries, and opinion-based papers, were also excluded. Grey literature was not included in this review.

Study Selection

There was a systematic and stepwise approach to the study selection. Study selection was conducted in accordance with the PRISMA guidelines. Initially, all identified records were screened based on titles and abstracts to exclude irrelevant studies. The remaining articles underwent full-text evaluation to determine eligibility according to predefined inclusion criteria. The study selection process is summarized using a PRISMA flow diagram. The rest of the studies were then evaluated using the full-text evaluation in order to establish their eligibility with the set inclusion criteria. It was at this point that any research that failed the test was eliminated. Such selection was conducted in a manner that only useful and quality studies could be included in the final review. It is an organized method of screening that minimizes bias and strengthens the credibility of the selected evidence to be evaluated.

Data Extraction

Data extraction was conducted using a predefined and standardized data extraction form developed for this review. The form was piloted on a subset of the included studies to ensure consistency, clarity, and completeness before full implementation. Extracted data included study characteristics (author, year, setting), study design, population details, type of intervention (e.g., media, educational, community-based), and key outcomes related to health literacy, awareness, and health-related behaviors.

Data extraction was performed systematically to ensure accuracy and consistency across studies. The extracted information was compiled into a structured summary table to facilitate comparison of study characteristics and findings. This approach enabled a comprehensive synthesis of evidence across different intervention types while maintaining methodological rigor.

Risk-of-Bias Assessment

The risk of bias of included studies was assessed using validated tools appropriate to study design. Randomized controlled trials were evaluated using the Cochrane Risk of Bias (RoB 2) tool, while observational studies were assessed using the Risk Of Bias In Non-randomized Studies of Interventions (ROBINS-I) tool. Campaign evaluation studies were appraised using adapted criteria focusing on potential confounding, selection bias, and outcome measurement reliability. Each study was assessed across relevant domains, including selection bias, performance bias, detection bias, attrition bias, and reporting bias. Based on these domain-level assessments, studies were categorized as having low, moderate, or high risk of bias. Randomized studies generally demonstrated lower risk due to controlled designs, whereas observational studies showed moderate risk, primarily due to confounding factors and reliance on self-reported data. This structured and design-specific approach ensured a consistent and transparent evaluation of study quality across heterogeneous study types.

Data Synthesis

Integration of findings from the included studies was conducted using a narrative synthesis approach. A quantitative meta-analysis was not feasible due to heterogeneity in study designs, interventions, and outcome measures. Instead, findings were categorized into themes using a deductive approach, based on predefined intervention types identified during the review design. These themes included (1) media and digital communication interventions and (2) community engagement and educational interventions. Within each theme, study findings were systematically compared to identify patterns, consistencies, and variations in outcomes related to health literacy, awareness, and behavior change. This structured approach facilitated meaningful cross-study comparisons and enabled interpretation of the relative effectiveness of different intervention types. The synthesis was conducted in a transparent and systematic manner to ensure coherence and interpretability of results. A meta-analysis was not conducted due to substantial heterogeneity in study designs, interventions, and outcome measures; therefore, a narrative synthesis was undertaken. Statistical pooling (e.g., P-values, confidence intervals) was not feasible. Themes were derived inductively through iterative coding of study findings.

Results

Study Selection

**Figure 1:**
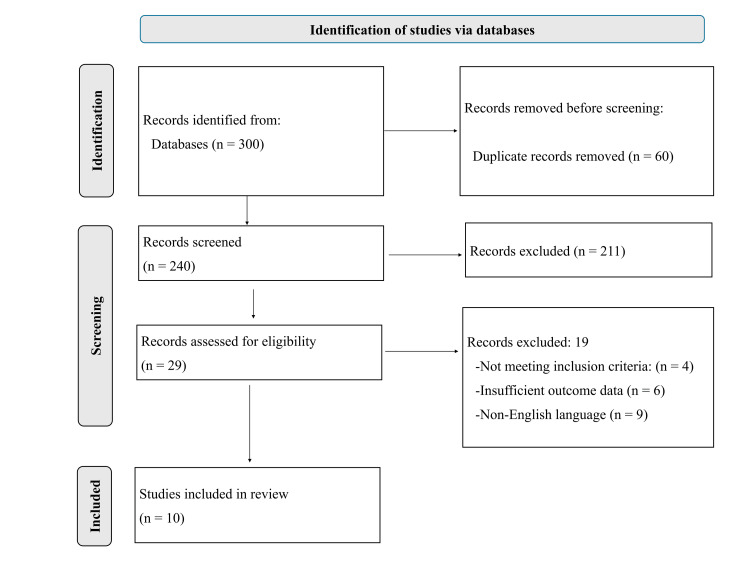
PRISMA 2020 flow diagram of study selection process based on searches conducted in PubMed, Scopus, and Google Scholar PRISMA, Preferred Reporting Items for Systematic Reviews and Meta-Analyses

A total of 300 records were identified through database searching. After removal of 60 duplicate records, 240 records were screened based on titles and abstracts, of which 211 were excluded due to irrelevance. Full-text assessment was conducted for 29 studies, resulting in the exclusion of 19 studies for reasons including not meeting inclusion criteria (n = 4), insufficient outcome data (n = 6), and non-English language (n = 9). Ultimately, 10 studies were included in the final analysis. The study selection process is illustrated in the PRISMA flow diagram (Figure 1).

Study Characteristics

The included studies (n = 10) comprised diverse methodological designs, including randomized controlled trials (n = 1), quasi-experimental studies (n = 1), observational studies (n = 3), comparative studies (n = 2), and campaign or population-level analyses (n = 3). Interventions were systematically categorized into two primary groups: (1) media and digital communication interventions (n = 6), including mass media campaigns, news/information exposure, and social media-based strategies, and (2) educational and community engagement interventions (n = 4), including video-assisted education, peer-led approaches, image-based awareness campaigns, and participatory models such as the SALT framework.

Media and digital interventions were predominantly implemented at national or large-scale levels and were mainly associated with changes in awareness, risk perception, and behavioral intentions, particularly in the context of smoking and infectious diseases. In contrast, educational and community-based interventions were more frequently linked to improvements in health literacy, increased participation in health-related activities, and adoption of preventive behaviors. Across the included studies, outcomes were most commonly reported as improvements in health literacy (n = 4), increased awareness or knowledge (n = 3), changes in attitudes and risk perception (n = 2), and behavioral change or preventive practice adoption (n = 4). These findings indicate a pattern in which communication-based interventions are more effective in influencing awareness and perceptions, whereas participatory and educational approaches contribute more consistently to sustained behavior change. The detailed characteristics of included studies are presented in Table 1.

**Table 1:** Characteristics of the included studies AOR, adjusted odds ratio; CI, confidence interval; COVID-19, coronavirus disease 2019; HL, health literacy; KAP, knowledge, attitude, and practice; RCT, randomized controlled trial; SALT, stimulate, appreciate, learn, transfer; SD, standard deviation

Study	Study Design	Setting / Location	Sample Size	Target Population	Intervention / Exposure	Duration of Intervention	Follow-up Period	Key Outcome(s)
Saei et al. [16]	Cross-sectional / evaluative study	Tehran, Iran (urban areas)	500	General adult population	Health channel broadcasting (mass media)	Not specified (exposure-based)	None	Improved health literacy and health behaviors
Hashemi-Shahri et al. [17]	Observational study	Iran (COVID-19 context)	Not specified	General community	News sources / information exposure	Not specified	None	Enhanced community health literacy regarding COVID-19
Abuhashesh et al. [18]	Comparative international study	Jordan and Poland	1149	General population / social media users	Social media use during COVID-19	Not specified	None	Increased public health awareness
Zhang et al. [19]	Quasi-randomized controlled trial	Shenzhen, China (schools)	2526	Primary school children	Video-assisted education + peer education	5 months (2 sessions)	~4 months	Improved health literacy on COVID-19 and infectious diseases
MacMonegle et al. [20]	Campaign evaluation study	United States	3354	Adolescents (11–16 years)	National anti-smoking media campaign	Ongoing exposure	~1 year	Changes in beliefs and perceptions about e-cigarettes and smoking
Aldukhail et al. [21]	Population-level analysis	United States	53,738	Adolescents (middle and high school)	“The Real Cost” campaign	2018–2020 cycles	Cross-sectional (repeated)	Increased risk perception and reduced curiosity about smoking
Duke et al. [22]	Longitudinal / campaign impact study	United States (75 media markets)	5103	Non-smoking adolescents	“The Real Cost” media campaign	2013–2016	Up to 3 years	Reduced smoking initiation
MacMonegle et al. [23]	Prospective / impact analysis	United States	3408	Adolescents (11–18 years)	“The Real Cost” campaign (e-cigarettes)	~1 year	~1 year	Reduced e-cigarette initiation
Makau-Barasa et al. [24]	Observational comparative study	Abuja, Nigeria	691	Community members	Image-based awareness + mobilization	Short-term campaign	Immediate / short-term	Increased participation, treatment uptake, and behavior change
Pramanik et al. [25]	Cluster randomized controlled trial	Assam, India (rural)	~180 villages (~2700 households)	Households with children (6–23 months)	SALT community engagement intervention	~1 year	Baseline to endline	Improved immunization coverage and community ownership

Risk-of-Bias Assessment

The overall risk of bias varied across studies depending on study design and methodological rigor. Randomized and quasi-randomized trials generally demonstrated lower risk of bias due to structured study designs, controlled allocation procedures, and systematic outcome assessment. However, some limitations were identified, including potential issues related to allocation concealment and consistency in intervention implementation.

Observational and cross-sectional studies were assessed as having a moderate risk of bias. This classification was based on several methodological limitations, including reliance on self-reported data, which introduces measurement bias; lack of control groups, limiting causal inference; and potential confounding factors that were not consistently adjusted for. Additionally, studies evaluating large-scale media campaigns faced challenges related to attribution, as external influences such as concurrent public health initiatives and environmental factors could affect observed outcomes. Given these limitations across multiple domains, including selection bias, confounding, and outcome measurement, the overall risk of bias for the body of evidence was considered moderate. A detailed summary of the risk of bias assessment for each study is presented in Table 2.

**Table 2:** Risk-of-bias summary

Study	Selection Bias	Performance Bias	Detection Bias	Attrition Bias	Reporting Bias	Overall Risk
Saei et al. [16]	Moderate	Moderate	Moderate	Low	Moderate	Moderate
Hashemi-Shahri et al. [17]	Moderate	Moderate	Moderate	Low	Moderate	Moderate
Abuhashesh et al. [18]	Moderate	Moderate	Moderate	Low	Moderate	Moderate
Zhang et al. [19]	Low	Moderate	Low	Low	Low	Low–moderate
MacMonegle et al. [20]	Moderate	Moderate	Low	Low	Moderate	Moderate
Aldukhail et al. [21]	Moderate	Moderate	Low	Low	Moderate	Moderate
Duke et al. [22]	Low	Moderate	Low	Low	Low	Low–moderate
MacMonegle et al. [23]	Low	Moderate	Low	Low	Low	Low–moderate
Makau-Barasa et al. [24]	Moderate	Moderate	Moderate	Low	Moderate	Moderate
Pramanik et al. [25]	Low	Low	Low	Low	Low	Low

Role of Media and Digital Communication in Public Health Awareness

**Figure 2:**
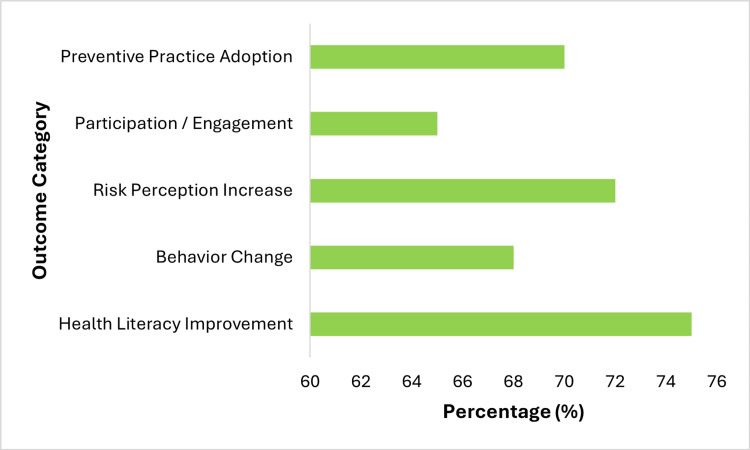
Media communication impact on health outcomes

The reviewed studies indicate that mass media and digital communication platforms play a significant role in increasing health awareness among populations. Conventional media and news outlets, alongside social media, have been shown to support the dissemination of health-related information and contribute to improvements in health literacy. These communication strategies were associated with increased risk awareness, enhanced understanding of health threats, and the development of preventive attitudes and behavioral intentions. Media campaigns, particularly those targeting younger populations, were linked to changes in attitudes toward harmful behaviors such as smoking and e-cigarette use. Similarly, digital platforms facilitated rapid and wide dissemination of health information during public health emergencies, supporting informed decision-making and adoption of preventive practices. Figure 2 illustrates the reported improvements across key health outcomes.

Effectiveness of Community Engagement and Educational Interventions

Community-based and educational interventions, including participatory models such as the SALT framework, image-based awareness campaigns, and structured educational approaches such as video-assisted and peer-led programs, were identified in four studies (n = 4) as key strategies for improving health outcomes, particularly in contexts requiring sustained behavioral change and active community involvement. These interventions emphasized local participation, community ownership, and context-specific implementation, which contributed to their observed effectiveness. Participatory approaches, such as the SALT model (n = 1), were associated with improved community engagement and increased uptake of health services, including immunization coverage. Image-based awareness campaigns (n = 1) were linked to higher participation and treatment uptake by presenting health information in a visually accessible and relatable manner. Educational interventions incorporating video-assisted learning and peer education (n = 1) demonstrated improvements in health literacy among school-aged populations. Across these studies, community engagement and educational interventions were associated with improvements in health literacy (n = 2), increased participation and service uptake (n = 2), and adoption of preventive health behaviors (n = 2).

**Figure 3:**
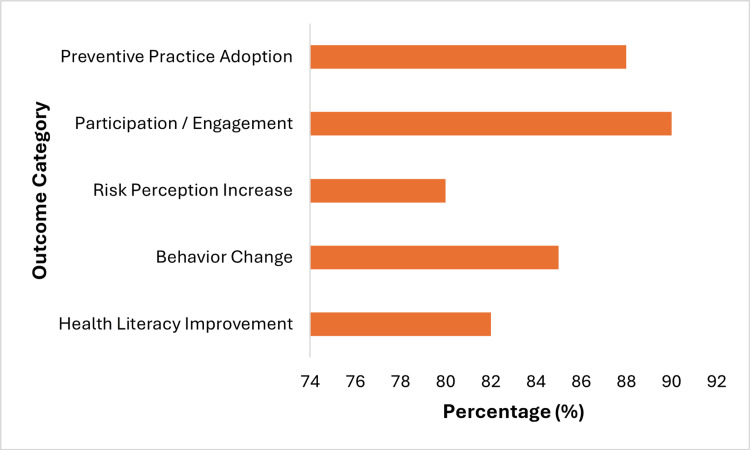
Community engagement impact on health outcomes

Participation and engagement outcomes were the most prominent (approximately 90%), followed by preventive practice adoption (approximately 88%) and behavioral change (approximately 85%). Improvements in health literacy were observed at around 82%, while increases in risk perception were comparatively lower (approximately 80%). These findings indicate that participatory and educational interventions are particularly effective in promoting engagement and translating awareness into sustained preventive behaviors, with relatively less impact on risk perception alone, as illustrated in Figure 3.

Discussion

The current systematic review synthesizes evidence on the effectiveness of communication-based and community engagement interventions in improving health literacy, awareness, and health-related behaviors. The findings indicate that media-based approaches and community participation play complementary roles in shaping population health outcomes. Media and digital communication interventions were consistently associated with improvements in awareness, risk perception, and behavioral intentions, particularly among younger populations; however, these effects appear to be context-dependent and are more pronounced in short-term or campaign-based interventions rather than sustained behavioral outcomes. These findings are consistent with prior studies demonstrating the central role of communication in influencing health behaviors and decision-making [26]. Previous studies have shown that mass media campaigns can effectively improve health knowledge and modify attitudes through repeated exposure and framing of health risks [27]; however, the long-term impact of such interventions on behavior remains variable. Similarly, the increasing reliance on online platforms reflects the evolving nature of health communication, where digital sources contribute significantly to shaping individual and social health behaviors [28], although their effectiveness depends on factors such as content credibility and user engagement. In contrast, community engagement and participatory interventions demonstrate stronger associations with sustained behavioral outcomes, including increased service uptake and adoption of preventive practices. This difference suggests a functional distinction between intervention types, where communication strategies primarily influence cognitive outcomes (awareness and perception), while participatory and context-specific approaches are required to translate these changes into sustained behavior. Overall, the findings highlight the importance of integrating communication-based and community-driven strategies to achieve both immediate and sustained public health outcomes.

Communication on health matters is increasingly facilitated through social media and digital platforms, particularly in the context of infectious disease awareness. From a theoretical perspective, these platforms align with interactive models of health communication and health literacy frameworks, which emphasize user engagement, feedback loops, and active information processing as key determinants of effective knowledge translation. Digital platforms enable users to interact with content, share information within networks, and seek clarification, thereby enhancing engagement and reinforcing message retention. Empirical evidence suggests that such interactivity is associated with measurable engagement outcomes, including increased information sharing, higher levels of participation in health discussions, and improved recall of health messages [7,9]. These mechanisms indicate that digital interventions are most effective when engagement is active rather than passive, highlighting the importance of user interaction in strengthening intervention impact. These engagement mechanisms contribute to improved awareness and short-term behavioral intentions, although their impact on sustained behavior change remains variable.

In the context of integrated interventions, combining digital communication with community-based and participatory approaches is theoretically supported by the complementary roles of information dissemination and social reinforcement. While digital platforms expand reach and engagement, community-based interventions provide contextual relevance and support for sustained behavior change. This synergy suggests that integrated interventions may be more effective than single-component strategies, as they simultaneously address both informational and behavioral determinants of health. However, practical implementation challenges remain, including variability in digital access, information credibility, and user engagement, which may influence the overall effectiveness of such integrated strategies. Previous studies also indicate that social media can support health literacy when content is accessible and tailored to target populations [29]. The spread of misinformation remains a significant limitation; however, structured and evidence-based communication strategies can mitigate these risks and enhance the credibility of health information [30].

Community involvement and educational interventions were associated with improvements in health-related behaviors, particularly in studies employing longitudinal and quasi-experimental designs that assessed outcomes over time. Evidence from these studies indicates that participatory approaches, such as the SALT model, which emphasize local ownership, community empowerment, and context-specific problem-solving, contribute to increased participation and improved uptake of health services. These findings are supported by both quantitative outcomes, such as increased immunization coverage and service utilization, and qualitative observations of enhanced community engagement and ownership. The effectiveness of these approaches is likely mediated by social reinforcement and contextual relevance, which strengthen the translation of knowledge into sustained behavioral practices. These findings align with theoretical frameworks emphasizing the role of social context, collective participation, and empowerment in shaping health behaviors [31].

Educational interventions, particularly those incorporating visual tools and peer-led methods, were effective in improving health literacy among specific populations such as school children. These approaches enhance comprehension by making health information more accessible and relatable. The role of eHealth literacy is also relevant, as increased reliance on digital platforms for health information is associated with improved decision-making capacity [32]. However, the effectiveness of these interventions may vary depending on baseline literacy levels and access to digital resources, indicating the need for tailored implementation strategies.

Another key finding of this review is the complementary role of media-based and community-based interventions. Media campaigns are consistently associated with improvements in awareness and risk perception at a population level, whereas community engagement approaches appear more effective in translating these changes into sustained behavioral outcomes. Existing literature suggests that integrating mass communication with locally implemented participatory interventions can enhance overall effectiveness and is associated with improved long-term outcomes; however, the strength of this evidence varies across study designs and contexts [33]. Despite these positive findings, several methodological limitations must be considered when interpreting the results. The included studies comprised a mix of experimental and observational designs, resulting in heterogeneity in intervention types, outcome measures, and evaluation methods. Observational studies are particularly susceptible to confounding and often rely on self-reported data, which may introduce measurement bias. In addition, studies evaluating large-scale media campaigns face challenges in attributing observed effects solely to the intervention, as external factors, such as concurrent public health initiatives or environmental influences, may also contribute to the outcomes. These limitations may lead to overestimation of intervention effectiveness, particularly in studies relying on self-reported outcomes and non-controlled designs. These limitations reduce the ability to establish causal relationships and should be considered when interpreting the reported effectiveness [28].

Variability in study contexts, including differences in population characteristics, cultural factors, and healthcare systems, further limits the generalizability of findings. As a result, the effectiveness of interventions may not be directly transferable across settings without contextual adaptation. These factors highlight the need for cautious interpretation and underscore the importance of culturally tailored and context-specific implementation strategies. The findings suggest that communication-based strategies are effective in influencing awareness and perceptions, while participatory approaches support sustained behavior change. However, given the methodological limitations and contextual variability, the evidence should be interpreted as indicative rather than definitive. Future research should prioritize longitudinal and rigorously designed studies to evaluate long-term outcomes and identify optimal combinations of communication and community-based interventions.

Limitations and Future Directions

Heterogeneity in study designs, interventions, and outcome measures represents a key limitation, restricting the comparability and synthesis of findings across studies. Several included studies relied on self-reported data, which may introduce recall bias and social desirability bias, potentially affecting the accuracy of reported outcomes. In addition, observational study designs are susceptible to selection bias and confounding, limiting the ability to establish causal relationships. The inclusion of only English-language studies may have introduced language bias and resulted in the exclusion of relevant evidence. Furthermore, variability in study settings, including differences in population characteristics, cultural contexts, and healthcare systems, limits the generalizability of findings across different contexts.

Well-designed randomized controlled trials should be focused in future research to enhance the quality of the evidence and the ability to inquire causally. Longitudinal research should be given a higher priority to determine the sustainability and long-term behavioral consequences. The combination of digital health communication with the model of community-based participation approaches should be systematically tested to find out the best intervention models. Consistency of results measures among studies would enhance comparability and facilitate the use of meta-analytical techniques. Second, the future research should explore culturally-specific interventions (at least in the case of low- and middle-income nations) to enhance cultural relevance. The issue of misinformation and its application in health communication also needs to be researched, and a method of improving credibility and trust in health communication needs to be designed.

## Conclusions

This systematic review demonstrates that communication-based interventions, particularly mass media and digital platforms, are consistently associated with improvements in health awareness, risk perception, and short-term behavioral intentions. In contrast, community-based and educational interventions are more strongly associated with sustained behavior change, increased participation, and improved uptake of health services. The findings indicate that no single intervention is sufficient to address complex public health challenges. Instead, integrated approaches that combine wide-reaching communication strategies with locally tailored, participatory interventions are more effective and are associated with both immediate and sustained outcomes. While media-based interventions enable rapid dissemination of information, community engagement approaches provide the contextual support necessary for long-term adoption of healthy behaviors. The evidence should be interpreted with caution due to methodological limitations, including heterogeneity in study designs, reliance on self-reported data, and potential biases. These factors limit causal inference and generalizability across settings. The review supports the use of multi-level, integrated public health strategies that combine communication and community engagement. Future research should focus on longitudinal and rigorously designed studies to evaluate long-term effectiveness and identify optimal combinations of interventions across diverse populations and contexts.
